# Biochemical Convergence of Mitochondrial Hsp70 System Specialized in Iron–Sulfur Cluster Biogenesis

**DOI:** 10.3390/ijms21093326

**Published:** 2020-05-08

**Authors:** Malgorzata Kleczewska, Aneta Grabinska, Marcin Jelen, Milena Stolarska, Brenda Schilke, Jaroslaw Marszalek, Elizabeth A. Craig, Rafal Dutkiewicz

**Affiliations:** 1 Intercollegiate Faculty of Biotechnology, University of Gdansk and Medical University of Gdansk, Abrahama 58, 80-307 Gdansk, Poland; malgorzata.nowak@phdstud.ug.edu.pl (M.K.); aneta.grabinska@phdstud.ug.edu.pl (A.G.); marcin.jelen@phdstud.ug.edu.pl (M.J.); milena.stolarska@phdstud.ug.edu.pl (M.S.); 2 Department of Biochemistry, University of Wisconsin, 433 Babcock Drive, Madison, WI 53706, USA; schilke@wisc.edu

**Keywords:** molecular chaperones, J-domain protein cochaperones, FeS transfer, gene duplication, protein evolution, yeast

## Abstract

Mitochondria play a central role in the biogenesis of iron–sulfur cluster(s) (FeS), protein cofactors needed for many cellular activities. After assembly on scaffold protein Isu, the cluster is transferred onto a recipient apo-protein. Transfer requires Isu interaction with an Hsp70 chaperone system that includes a dedicated J-domain protein co-chaperone (Hsc20). Hsc20 stimulates Hsp70′s ATPase activity, thus stabilizing the critical Isu–Hsp70 interaction. While most eukaryotes utilize a multifunctional mitochondrial (mt)Hsp70, yeast employ another Hsp70 (Ssq1), a product of mtHsp70 gene duplication. Ssq1 became specialized in FeS biogenesis, recapitulating the process in bacteria, where specialized Hsp70 HscA cooperates exclusively with an ortholog of Hsc20. While it is well established that Ssq1 and HscA converged functionally for FeS transfer, whether these two Hsp70s possess similar biochemical properties was not known. Here, we show that overall HscA and Ssq1 biochemical properties are very similar, despite subtle differences being apparent - the ATPase activity of HscA is stimulated to a somewhat higher levels by Isu and Hsc20, while Ssq1 has a higher affinity for Isu and for Hsc20. HscA/Ssq1 are a unique example of biochemical convergence of distantly related Hsp70s, with practical implications, crossover experimental results can be combined, facilitating understanding of the FeS transfer process.

## 1. Introduction

Iron–sulfur cluster(s) FeS are ancient cofactors required for activity of many proteins functioning in all cellular compartments. FeS proteins are necessary for critical cellular processes such as oxidative respiration, photosynthesis, nitrogen fixation and DNA replication/repair. Multiprotein machineries dedicated to FeS biogenesis are present in all domains of life. In eukaryotes, mitochondria, which inherited most of the proteins involved in FeS biogenesis from their bacterial ancestors, play a central role in this process [1,2]. Both bacterial and mitochondrial FeS biogenesis pathways consist of two major steps: First, de novo assembly of clusters on a dedicated scaffold protein (termed Isu and IscU in eukaryotes and bacteria, respectively); Second, cluster transfer from the scaffold onto recipient apo-proteins. In most proteobacteria and in mitochondria, Heat shock protein 70 (Hsp70) molecular chaperones plays a critical role in the FeS transfer step [3,4,5,6]. 

In general, Hsp70 function depends on cyclic interaction with protein substrate, Isu/IscU in the case of FeS biogenesis, that is regulated by conformational changes of Hsp70 induced by ATP binding and hydrolysis [7]. Hsp70s do not function alone, a J-domain protein (JDP) co-chaperone plays a key role in this interaction cycle—its signature J-domain transiently interacts with Hsp70, stimulating its adenosine triphosphate-ase (ATPase) activity, thereby triggering Hsp70 conformational changes that stabilize substrate interaction [8,9,10,11]. In FeS biogenesis, a dedicated JDP co-chaperone, termed Hsc20 (often called HscB in bacteria) is key, binding cluster-loaded Isu on its own and targeting it for the Hsp70 binding [6]. Evidence suggests that this interaction is a key for the FeS transfer onto recipient apo-protein [12,13,14]. The interaction cycle of all Hsp70-substrate interactions is completed by adenosine diphosphate (ADP) exchange for ATP, which induces another Hsp70 conformational change, resulting in the release of its substrate (Isu/IscU in the case of FeS biogenesis). ADP/ATP exchange in most systems requires assistance of a dedicated cochaperone protein termed nucleotide exchange factor (NEF) [15].

Though Hsc20 and Isu (paralogous Isu1 and Isu2 scaffolds in *S. cerevisiae*) are direct descendants of bacterial HscB and IscU, respectively, Hsp70s involved in the FeS biogenesis have a complex evolutionary history [16,17,18,19]. In *Escherichia coli*, similar to other bacteria, the highly specialized HscA has a single protein substrate, the IscU scaffold protein [20,21]. Moreover, HscA, in contrast to other Hsp70s, including the major bacterial Hsp70 termed DnaK, does not required a NEF for the ADP/ATP exchange [6,22]. In contrast, mitochondria of most organisms, including humans, do not have an Hsp70 specialized in the FeS biogenesis [17]. Rather, Hsc20 targets cluster loaded Isu to the multifunctional mitochondrial Hsp70 (mtHsp70)—the only Hsp70 present—and thus functioning in many other critical processes, such as de novo protein folding and refolding after stress, as well as protein import [7]. Thus, mtHsp70 must cooperate with several different mitochondrial JDPs for interaction with a multitude of different protein substrates, including with Hsc20 for Isu interaction. mtHsp70, similar to bacterial DnaK, requires a NEF, termed Mge1, for the ADP/ATP exchange during the substrate binding cycle [23]. 

Mitochondria of *S. cerevisiae* and related yeast species contain a second Hsp70 termed Ssq1. Ssq1 is a product of a mtHsp70 gene duplication that took place at the base of the *S. cerevisiae* lineage and became specialized in FeS cluster biogenesis [17]. Ssq1 cooperates only with Hsc20 and binds Isu exclusively, while its paralogue, termed Ssc1, maintained multifunctionality typical of the ancestral mtHsp70 [24,25]. Both Ssq1 and Ssc1 require Mge1 for ADP/ATP exchange [26], like their mtHsp70 ancestor. Due to this specialization, *S. cerevisiae* has become a favorite model organism for researchers studying the FeS cluster biogenesis in mitochondria. Powerful yeast genetics and cell biology methods, combined with the unique presence of the specialized Ssq1/Hsc20 system, have allowed a number of questions related to the overall organization of the FeS biogenesis pathway to be answered [1,3].

While it is well established that these chaperone systems have converged functionally to specialize in FeS biogenesis, neither the evolutionary relationships between them [16,17], nor the extent of the similarities/differences between their biochemical properties [3,6] have been systematically studied. This is an important point, because a mechanistic understanding of the role of the Hsp70 system in the mitochondrial process of cluster biogenesis is rather limited [1,4]. This gap is due to difficulties in reconstituting the FeS transfer process with purified proteins, as Ssq1, Hsc20 and Isu can be purified only in limited quantities. On the other hand, their bacterial equivalents, HscA/HscB and IscU can be easily purified in high quantities, allowing detailed biochemical analyses [5,6]. Therefore, the extent to which biochemical results obtained for the bacterial HscA/HscB system are transferable for understanding mitochondrial processes is unclear, mainly because these systems have been studied in different laboratories using different experimental protocols. 

In this study, we have clarified evolutionary relationships between bacterial and mitochondrial Hsp70s specialized in the FeS biogenesis, demonstrating that Ssq1 is more closely related to DnaK than to HscA. However, side-by-side comparisons of HscA and Ssq1 systems revealed that their biochemical properties are remarkably similar despite their divergent evolutionary origins. Thus, these systems provide a unique example of convergent evolution at the biochemical level. Moreover, our results can help in unraveling mechanistic details of the FeS biogenesis by allowing the combining of results obtained from two specialized systems in an informative way. 

## 2. Results

### 2.1. HscA Is Distantly Related to Ssq1 and Not Present in Eukaryotic Proteomes

Despite years of research, the evolutionary relationships between HscA/HscB and Ssq1/Hsc20 systems has not been fully resolved [16,17,19]—even the key question of whether HscA is present only in bacteria or whether its orthologs are also present in eukaryotes. To more precisely define the evolutionary relationship between HscA and Ssq1, we reconstructed the protein phylogeny of their orthologs from a number of bacterial and eukaryotic species. Moreover, we used a large dataset of eukaryotic proteomes to test for the presence of HscA orthologs. 

**Figure 1:**
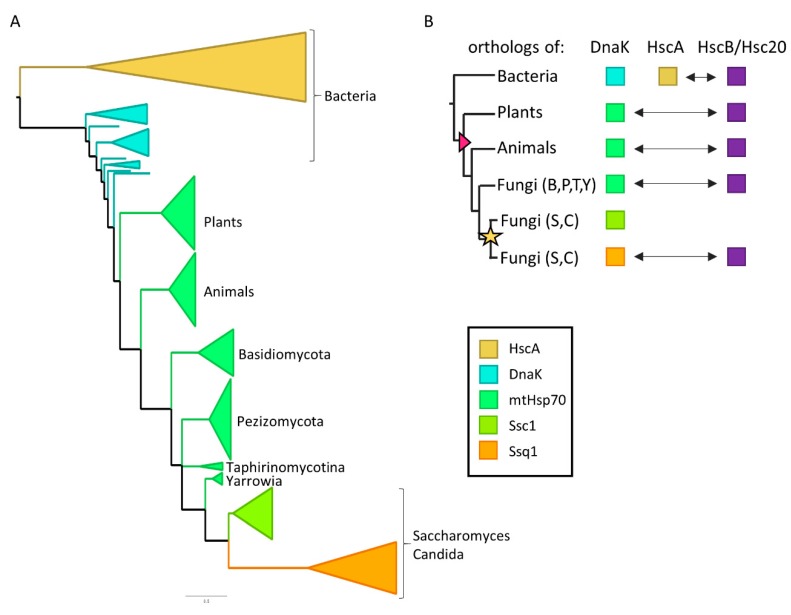
Evolutionary relationships among Hsp70 systems involved in the FeS biogenesis in bacteria and mitochondria. (**A**) Maximum likelihood phylogeny of Hsp70s involved in the FeS biogenesis. Scale bar: amino acid substitutions per site. Only nodes with bootstrap support >0.7 are resolved. The tree was rooted at the HscA and DnaK common ancestor for the clarity only. For fully resolved tree see Supplementary Figure S1. (**B**) Evolutionary history of Hsp70 systems involved in the FeS biogenesis. In bacteria specialized Hsp70 HscA functions in FeS biogenesis with dedicated JDP HscB, while multifunctional Hsp70 DnaK plays other biological roles. At the base of the Eukaryote lineage (pink triangle) HscA was lost. Cooperating with ortholog of HscB termed Hsc20, mtHsp70, a descendant of DnaK, replaced it in mitochondrial FeS biogenesis. At the base of the *Saccharomyces* and *Candida* clade (S,C), duplication of the mtHsp70 gene (yellow star) resulted in formation of Ssq1, which subsequently became specialized in FeS biogenesis, recapitulating specialized HscA/HscB bacterial system. Arrows indicate functional interactions between orthologs of HscB/Hsc20 and their Hsp70 partners. Fungi: Basidiomycota (B), Pezizomycotina (P), Taphrinomycotina (T), *Yarrowia* (Y), *Saccharomyces* (S), *Candida* (C).

The maximum likelihood phylogeny we obtained (Figure 1 and Supplementary Figure S1) indicates that mtHsp70, is more closely related to bacterial DnaK than to HscA, as HscA and mtHsp70 sequences are not monophyletic. Rather, mitochondrial Hsp70s form a monophyletic group with DnaK from Alphaproteobacteria, suggesting their endosymbiotic acquisition from the mitochondrial ancestor [27]. Mitochondrial Hsp70s found in mitochondria form a clade, which contains both multifunctional mtHsp70 and specialized Ssq1 proteins. This grouping of Ssq1 as sister to Ssc1 agrees with previously published data [16,17] indicating that Ssq1 emerged from a mtHsp70 gene duplication that took place at the base of the *Saccharomyces* and *Candida* clade. 

Our analysis of the distribution of HscA and HscB orthologs in eukaryotic proteomes using Hidden Markov Models (HMMs) based on bacterial sequences revealed the ubiquitous presence of Hsc20 orthologs in the 1151 eukaryotic proteomes tested, which is consistent with their essential role in the FeS biogenesis (Supplementary Figure S2A). In stark contrast, we conclude that there are no HscA orthologs in eukaryotic proteomes. Only 9 potential orthologs were identified with HMM profile search for HscA—7 of which clustered with DnaK and 2 with mtHsp70 when placed on the Hsp70 phylogeny (Figure S2B). Furthermore, BLAST searches revealed that all 9 are more similar to DnaK than to HscA (Table S1). Taken together, our data indicate that Ssq1 is more closely related to DnaK than to HscA and that HscA was lost during evolution of mitochondria (see Figure 1B for details).

### 2.2. HscA and Ssq1 Have Comparable Maximally Stimulated ATPase Rates

**Figure 2:**
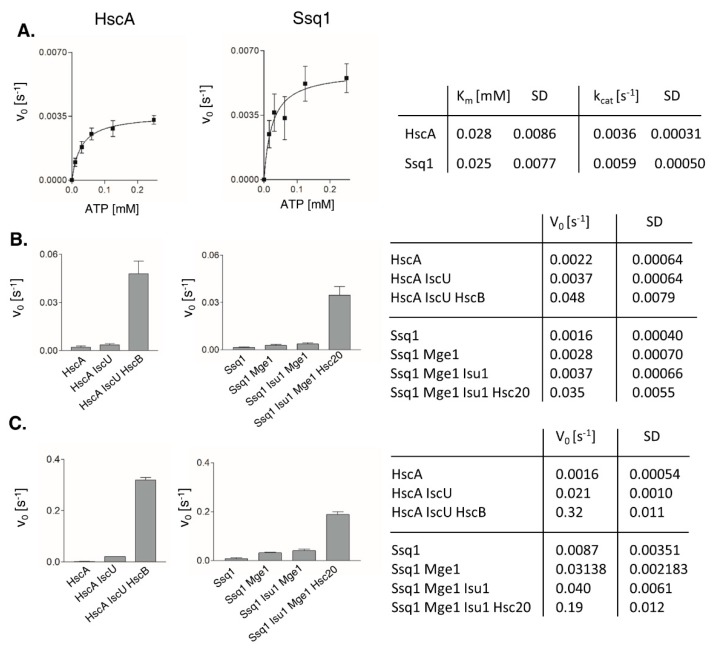
ATPase activities of HscA and Ssq1. (**A**) Affinity of HscA and Ssq1 for ATP. Reaction mixtures contained 1 μM HscA or 2.5 μM Ssq1 and Mge1. ATPase rates were measured at various ATP concentrations using an enzyme-coupled assay. Curves are the best fits of the data to the Michaelis–Menten equation. Error bars represent SD for three independent measurements. (**B**,**C**) Stimulation of HscA and Ssq1 ATPase activities in the presence of protein substrates and JDP cochaperones. Average ATPase rates for three independent measurements with error bars as SD are shown at left, and in table form at right. (**B**) The reaction mixtures contained 120 μM ATP, 0.5 μM HscA, 2 μM HscB, 10 μM IscU or 0.5 μM Ssq1, 2 μM Hsc20, 10 μM Isu1 and 0.5 μM Mge1. The release of radioactive inorganic phosphate from [γ−^33^P] ATP was measured. (**C**) The reaction mixtures contained 1 mM ATP, 0.5 μM HscA, 50 μM HscB, 50 μM IscU or 0.5 μM Ssq1, 50 μM Hsc20, 50 μM CtIsu1 and 0.5 μM Mge1. Release of inorganic phosphate was measured using the EnzChek assay.

To initiate biochemical comparisons, we analyzed CD spectra and melting temperatures (T_M_) of purified HscA, HscB, Ssq1 and Hsc20 proteins (Supplementary Figure S3); no significant differences were detected. Next, we measured affinities of Ssq1 and HscA for ATP. We used steady state ATPase assay performed in the presence of increasing concentrations of ATP, as obtained Km values can be taken as an approximate measure of ATP affinity. Because of the difficulty in measuring the low basal ATPase activities of Hsp70s, we used an enzyme coupled assay in which ATP hydrolyzed by an Hsp70 is coupled via a phosphoenolpyruvate (PEP)/pyruvate kinase (PK) regeneration system to the oxidation of reduced nicotinamide adenine dinucleotide NADH via L-lactate dehydrogenase (LDH). Moreover, for Ssq1, to avoid limiting the reaction rate by slow ADP/ATP exchange, measurements were conducted in the presence of nucleotide exchange factor Mge1. The obtained Km values for ATP were similar, 25 and 28 μM for Ssq1 and HscA respectively (Figure 2A), indicating similar affinities for ATP. 

Next, we compared the basal ATPase rates of Ssq1 and HscA. We used the same reaction conditions in an [γ-^33^P]-ATP based assay that we used previously for Ssq1 [23]. Basal ATPase rates were very similar for Ssq1 and HscA, respectively (Figure 2B). That the rates were similar was not surprising, as these low values are comparable to those of other members of the Hsp70 family [28,29]. ATPase activities increased only about 2-fold in the presence of protein substrate (IscU for HscA; Isu1 for Ssq1, plus NEF Mge1). Only when reaction mixtures contained both protein substrates and co-chaperones (Figure 2B), were ATPase rates dramatically higher (0.035 ± 0.0055 s^-1^ for Ssq1 and 0.048 ± 0.0079 s^−1^ for HscA). However, the small, 30% difference in the maximally stimulated ATPase rates for Ssq1 and HscA was unexpected, as about 10-fold higher ATPase rates for HscA in the presence of IscU and HscB were previously reported [20,30]. However, higher concentrations of IscU and HscB were used in these experiments. We therefore decided to compare Ssq1 and HscA ATPase rates using the previously published experimental conditions: 50 μM Isu/IscU and 50 μM Hsc20/HscB. Also, to make these measurements comparable to previously published data we used the commercially available EnzChek assay kit [20]. As we were not able to purify *S. cerevisiae* Isu1 at high enough concentrations, the thermophile fungus *Chaetomium thermophilum* ortholog (CtIsu1) was used in its place. We demonstrated previously that CtIsu1 stimulates the ATPase of Ssq1 very efficiently [19]; it has also been shown that CtIsu1 can replace native Isu1 in *S. cerevisiae* [31] *in vivo*. Under these conditions, the ATPase rates of HscA and Ssq1 were about 6-fold higher than those measured in the radioactive assay, 0.32 ± 0.011 s^−1^ compared to 0.19 ± 0.012 s^−1^, (Figure 2C). However, the difference between maximally stimulated HscA and Ssq1 ATPase rates was 40%, only slightly changed from the 30% measured in the radioactive assay. Taken together, our side-by-side measurements indicate that, although the fully stimulated ATPase rate of HscA is higher than those of Ssq1, the difference is not as dramatic as it could have been expected based on the previously published data [6,14,20,30]. 

### 2.3. Ssq1 Has Higher Affinity for Protein Substrate Than HscA

**Figure 3:**
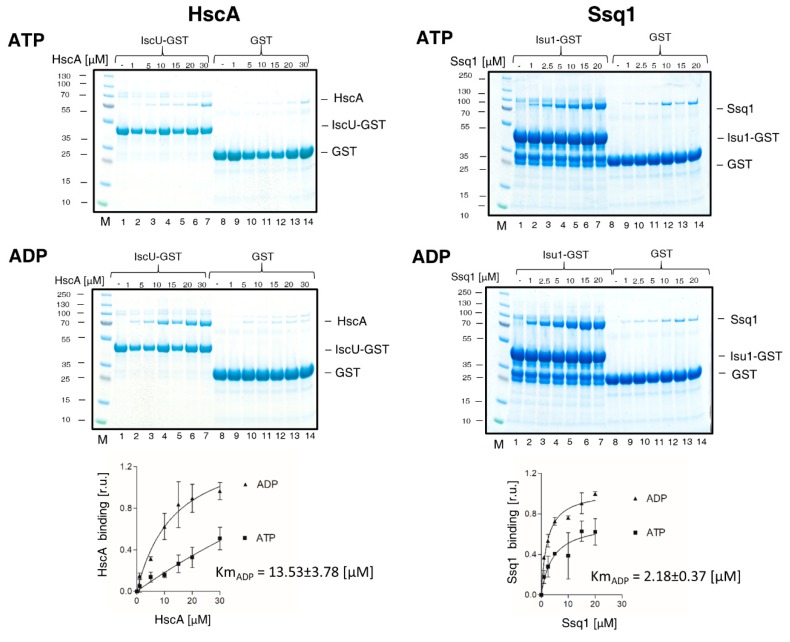
Affinity of HscA and Ssq1 for protein substrate (IscU/Isu1) in the presence of ATP or ADP. (**left**) IscU-GST (15 μM) or GST (15 μM) were incubated in the presence of ATP or ADP (4 mM) with increasing concentrations of HscA, as indicated. Glutathione resin was added to pull-down GST and associated proteins, which were then separated by sodium dodecyl sulfate-polyacrylamide gel electrophoresis (SDS-PAGE) and stained with Coomassie Blue. “M” indicates lanes having molecular weight markers; controls of loading are shown in Supplementary Figure S4. Bound HscA was quantitated by densitometry. Values were plotted as relative units (r.u.) with maximal signal for HscA set as 1. Curves represent best fit of the data to the Michaelis-Menten hyperbolic equation. The Km value obtained in the presence of ADP (Km_ADP_) is listed. Error bars represent SD for three independent measurements. (**right**) Isu1-GST or GST (30 μM) were incubated in the presence of ATP or ADP (4 mM) with increasing concentrations of Ssq1. Procedure was as described above for HscA/IscU.

It is well documented that Ssq1 and HscA interact very efficiently with their substrate, but how different/similar their affinities are is not known [21,23]. For a side by side comparison we used fusions between Isu1/IscU and glutathione S-transferase (GST), to take advantage of GST’s ability to bind glutathione [32]. Increasing concentrations of Ssq1/HscA were incubated with a fixed concentration of Isu1/IscU-GST fusion protein to allow complex formation; then glutathione resin was added to pull down GST fusion protein and any Hsp70 bound to it. We performed assays in the presence of ATP or ADP, as it is well documented [9,11] that in the ATP bound state the Hsp70 substrate binding cleft is “open” and high on and off rates result in low substrate affinity, while in the ADP bound state the substrate binding cleft is closed, resulting in high substrate affinity. The results obtained for both Ssq1 and HscA are consistent with these expectations. Efficient Isu1/IscU-GST binding was observed for both Hsp70s in the presence of ADP, while less efficient binding was observed in the presence of ATP (Figure 3). Km values in the presence of ADP were 2.18 ± 0.37 μM for Ssq1 and 13.53 ± 3.78 μM for HscA. As, the Km value obtained for the ADP bound state is a convenient measure of Hsp70’s affinity for protein substrate, by this measure, Ssq1 has a ~6-fold higher affinity for substrate than HscA. 

### 2.4. Ssq1 Has Higher Affinity for JDP Cochaperone Than HscA

The affinity of Hsp70 for its JDP partner is difficult to measure, because their interaction is very transient [33]. However, we previously developed [32] two assays allowing us to determine the apparent Km, an approximate measure of the affinity, of Hsc20 interacting with Ssq1. In one assay, we examined the stimulation of Ssq1 ATPase activity by titrating Hsc20 in the presence of excess Isu1. The other method is based on the Isu1-GST pull down assay described above. When the pull-down assay is executed in the presence of ATP the efficiency of the Ssq1/Isu1-GST interaction increases with the increasing concentrations of Hsc20, thus, allowing determination of its apparent Km. While the ATPase assay depends on the presence of Mge1 in the case of Ssq1, the pull-down assay does not, and therefore is particularly well suited for side by side comparison of the Ssq1/Hsc20 and HscA/HscB systems. 

**Figure 4:**
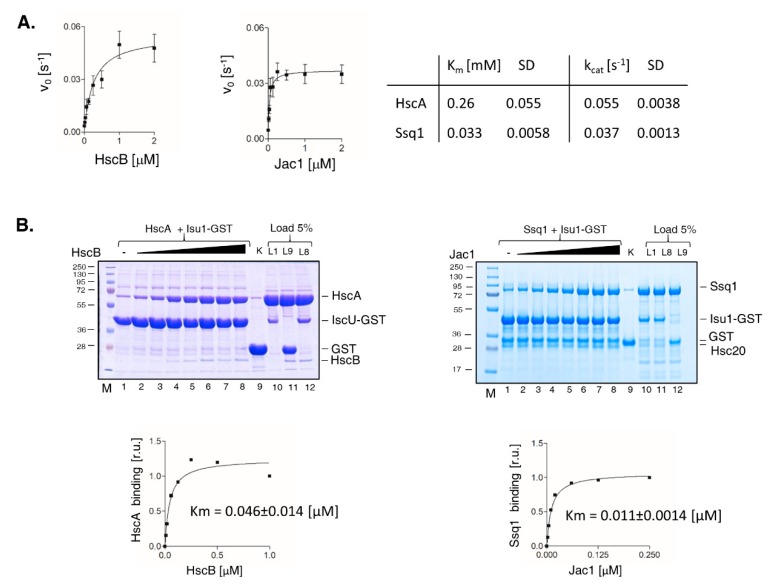
Affinity of HscA/Ssq1 for HscB/Hsc20 cochaperones. (**A**) Stimulation of HscA/Ssq1 ATPase activity at increasing concentrations of cochaperones. ATPase activities were measured with radioactive assay as described in Figure 2B but with increasing concentrations of HscB or Hsc20, as indicated. Curves represent best fit of the data to the Michaelis-Menten equation. Error bars represent SD for three independent measurements. (**B**) Stimulation of HscA/Ssq1 interaction with IscU/Isu1-GST in the presence of cochaperones and ATP. (**left**) IscU-GST (30 μM) was incubated with HscA (30 μM) in the presence of ATP (4 mM) and increasing concentrations of HscB (0.01; 0.02; 0.06; 0.125; 0.25; 0.5; 1 μM). Glutathione resin was added to pull-down GST and associated proteins, which were separated by SDS-PAGE and stained with Coomassie Blue. Lane 9: a control where IscU-GST was replaced by GST and 1 μM HscB + 30 μM HscA were present in the reaction mixture (“K”). Lanes 10-12: Five percent of the reaction mixtures that were run in lanes 1, 9 and 8, were run as loading control (Load 5%: L1, L9, L8). M: lane having molecular weight markers. Bound HscA was quantitated by densitometry. Values were plotted as relative units (r.u.) with maximal signal for HscA set at 1. Curve represent best fit of the data to the Michaelis-Menten equation. (**right**) Isu1-GST (2.5 μM) was incubated with Ssq1 (4 μM) in the presence of ATP (4 mM) and increasing concentrations of Hsc20 (0.0025; 0.005; 0.01; 0.02; 0.06; 0.125; 0.25 μM). Glutathione resin was added to pull-down GST and associated proteins, which were separated by SDS-PAGE and stained with Coomassie Blue. Lane 9: control where Isu1-GST was replaced by GST and 0.25 μM Hsc20 + 4 μM Ssq1 were added to the reaction mixture (“K”). Lanes 10–12: Five percent of the reaction mixtures run in lanes 1, 8 and 9, were run as loading controls (Load 5%: L1, L8, L9). M: lane having molecular weight markers. Bound Ssq1 was quantitated by densitometry. Values were plotted as relative units (r.u.) with maximal signal for Ssq1 set at 1. Curve represent best fit of the data to the Michaelis-Menten equation.

The apparent Km values determined by the ATPase assay for Hsc20 and HscB (Figure 4A) revealed the Ssq1’s affinity for Hsc20 to be about 8-fold higher than those of HscA for HscB. However, the k_cat_ measured in this assay was about 30% higher for HscA ATPase than for Ssq1, consistent with the results presented on Figure 2. To combine these two parameters in a single measure that represents the efficiency of the Hsp70/cochaperone interaction we calculated the k_cat_/Km ratio. The k_cat_/Km ratio was 1.21 s^−1^•μM for Ssq1 and 0.21 s^−1^•μM for HscA. Thus, also by this measure the efficiency of the Ssq1/Hsc20 system was about 5-fold higher than that of the HscA/HscB system. 

The apparent Kms obtained for Hsc20 and HscB using the pull-down assay, were lower (Figure 4B), than those obtained using the ATPase assay. But the overall trend was the same - the apparent Km for Hsc20 was ~4-fold lower than that for HscB. Taken together these results indicate that both Ssq1 and HscA interact with their JDP partners very efficiently and are able to stimulate their ATPase dependent substrate binding cycles at sub stoichiometric concentrations. It is worth noting, however, that in both assays newly evolved Ssq1 had higher affinity for Hsc20 than HscA had for HscB. 

## 3. Discussion

The results of our analyses clarify several issues concerning the evolutionary relationship among Hsp70 chaperones involved in mitochondrial and bacterial FeS biogenesis. They support a scenario in which bacterial HscA was lost during evolution of mitochondria and its role in the FeS biogenesis was taken over by mtHsp70, a descendant of bacterial multifunctional Hsp70 DnaK. Moreover, the results of our phylogenetic reconstruction support a notion that specialized Ssq1 is a product of mtHsp70 gene duplication that took place at the base of the *S. cerevisiae* lineage. These evolutionary scenarios have been discussed previously [16,18,25], but were based on the limited datasets available at that time, rather than the large data sets used in the analyses presented here. Based on our more complete results, no doubt remains: mitochondrial Ssq1 and bacterial HscA are distantly related and thus constitute a bona fide example of functional convergence—making the question of how similar or different these two Hsp70s are biochemically highly relevant from an evolutionary perspective [34,35,36]. 

A major finding of our comparative analyses of Ssq1 and HscA chaperones is the striking similarity of the two systems’ biochemical properties. ATPase activities of the two Hsp70s are stimulated to comparable levels by their JDP cochaperones (Hsc20/HscB) and protein substrates (Isu1/IscU), regardless of whether present at low, more physiologically relevant, or very high concentrations. Although the ATPase activity of Ssq1 is stimulated to a somewhat lower extent than that of HscA, the efficiency of the Ssq1 system, measured as the k_cat_/Km ratio, is higher than the HscA system, suggesting that at low concentrations of cochaperones and substrates the specialized mitochondrial system is more efficient than its bacterial counterpart. This suggests that the lower maximally stimulated ATPase rates observed for Ssq1 system can be compensated by its higher affinity for both Hsc20 and Isu1, consistent with the fact that ~10 % of the normal Hsc20 level is sufficient for normal growth [37]. Although the absolute cellular concentrations of proteins constituting the Ssq1 and HscA chaperone systems are not known, the relative values available in the literature suggest that Ssq1 and Hsc20 are present at close to equimolar amounts, in the micromolar concentrations range; the same is true for HscA and HscB [https://pax-db.org/]. Thus, similar biochemical properties of these two specialized systems, which we are postulating here, are most likely physiologically relevant.

How do the specialized systems discussed here compare to the multifunctional mtHsp70 functioning in most mitochondria in FeS biogenesis, including humans? Although little is known about the multifunctional system, it is clear that mtHsp70 is highly abundant. In *S. cerevisiae,* Ssc1 is ~1000 fold more abundant than Ssq1, making up ~2% of total mitochondrial proteins [24]. Also, the human mtHsp70 (HSPA9) is a highly abundant mitochondrial protein [https://pax-db.org/]. Furthermore, in an analysis of *Schizosaccharomyces pombe*, which has no specialized Hsp70, Hsc20 was found to have a ~100 fold lower affinity for mtHsp70 than *S. cerevisiae* Hsc20 has for Ssq1 or *E. coli* HscB has for HscA [19]. This large difference suggests that specialized and multifunctional Hsp70 systems use very different biochemical strategies. In the case of specialized Hsp70 systems, high affinities for Hsc20 cochaperones enables them to operate at low concentrations. While, in the case of multifunctional Hsp70s, their low affinity for Hsc20 cochaperones is most likely compensated for by very high abundance. Why have mtHsp70s not evolved a higher affinity for Hsc20? Most likely because of its multifunctionality. As different functions require cooperation of mtHsp70 to cooperate with different JDPs, JDPs are in fact competing with one another for access to the same mtHsp70 partner [7]. This competition between JDPs prevents any of them from evolving higher affinity toward mtHsp70, as it would compromise other mitochondrial functions. Further studies are needed however to rigorously test this idea. 

Finally, the results presented here have important practical implications. The two specialized systems are favorite models for researchers studying mechanistic aspects of Hsp70′s involvement in the process of FeS biogenesis. However, the systems are better suited for different types of research. While the bacterial proteins are easy to purify in high quantities suitable for biochemical studies, powerful genetics and cell biology tools make the yeast system an ideal model for in vivo research. Our demonstration that under physiologically relevant conditions these two systems are biochemically very similar should help researchers to use these two systems more effective to gain a better overall understanding of the important process of FeS cluster biogenesis. 

## 4. Materials and Methods

### 4.1. Evolutionary Analyses

Hsp70 sequences orthologous to mtHsp70, DnaK, HscA and cytosolic Hsp70 Ssa1 were retrieved from OMA orthology database. Sequences were aligned using Clustal Omega v1.2.2 with default parameters [38]. To infer phylogeny, multiple sequence alignments were converted into a Hidden Markov Model [39] using hmmbuild program from the HMMER package [40]. Forward–backward algorithm was used to compute a posterior probability (pp) for each site representing the degree of confidence in each position (residue or gap) of the alignments for each sequence. Amino acid positions with pp < 0.7 were removed from the multiple sequence alignment [41]. 1000 maximum likelihood (ML) searches were performed using IQ-tree [42] with 1000 ultrafast bootstrap replicates, under the LG model of amino acid substitution and GAMMA model of rate heterogeneity with four discrete rate categories and the estimate of proportion of invariable sites (LG + I + G), which was determined by IQ-tree as the best-fit model. 

Proteomes of 339 animal, 661 fungal, 113 plant and 38 protist species were retrieved from the UniProt database [43]. To search for HscA and HscB orthologs in collected proteomes one to one orthologs of HscA and HscB were retrieved from the OMA database and aligned using Clustal Omega v1.2.2 with default parameters. Both alignments were converted into Hidden Markov Models (HMM) using hmmbuild program. Proteome searches were performed using hmmsearch program from the HMMER package. First, the search algorithm was trained to recognize HscA, but not other Hsp70s using sequences from Figure 1 as a reference dataset, as described in [44] (similarity scores > 853). Second, the search algorithm was trained to recognize HscB, but not other J-domain proteins (JDP) using 135 bacterial JDP proteins as a reference dataset (similarity score > 38). Hits identified as potential HscA orthologs were further verified using a phylogenetic approach (see Figure S2B, Table S1). In this approach sequences were aligned with the Hsp70 reference dataset and the RAxML likelihood-based placement algorithm [45] was used to add them to the reference Hsp70 tree (Figure 1). Sequences grouping with the HscA clade were recognized as true positives, while sequences grouping outside the HscA clade were assigned as potential false positives. The latter were additionally verified using reciprocal BLAST searches to the *E. coli* proteome. 

### 4.2. Protein Purification

HscA and HscB proteins were purified as described by Vickery et al. 1997 [46] with some modifications. DH5α cells transformed with either pTrcHscA or pTrcHscB were grown in Terrific Broth at 37 °C, induced with 0.5 mM isopropyl β-D-1-thiogalactopyranoside (IPTG) at *A*_600_ = 1, and grown ~16 h to allow expression. Cells were harvested by centrifugation, frozen, thawed, and lysed by French press in Q1 buffer (50 mM Tris-HCl, pH 7.4, 1 mM dithiothreitol (DTT), 0.5 mM Ethylenediaminetetraacetic acid (EDTA),) containing 0.4 mM phenylmethylsulfonyl fluoride (PMSF) to inhibit proteolysis. The soluble supernatant fluid following centrifugation at 75,000× *g* was used for purification, and all subsequent chromatography was carried out in Q1 buffer at 4 °C. Cell lysates containing either HscA or HscB were initially purified using Q-sepharose (GE Healthcare, Uppsala, Sweden). Soluble extracts were applied to the column and washed with several column volumes of Q1 buffer containing 0.4 mM PMSF. HscA or HscB was eluted using a linear gradient of 0 to 1 M NaCl in buffer Q1, and fractions were analyzed by gel electrophoresis, then combined and concentrated using Amicon Ultra-15 Centrifugal Filter Units (Merck Millipore, Tullagreen, Cork, Ireland) and dialyzed against P1 buffer (100 mM Tris-HC1, pH 7.4, 1 mM DTT). After dialysis, a sample of HscA or HscB was supplemented with ammonium sulfate to 1 M concentration and applied to a Phenyl Sepharose (GE Healthcare, Uppsala, Sweden) column. HscA or HscB was eluted with a linear gradient decreasing from 1 M to zero ammonium sulfate. Fractions containing HscA or HscB were concentrated by ultrafiltration and subjected to buffer exchange to buffer F (20 mM Tris-HCl pH 8.0, 20 mM KCl, 10% glycerol) using PD10 column (GE Healthcare, UK). The final preparation of HscA or HscB was aliquoted, frozen in liquid nitrogen, and stored at −70 °C.

Expression of IscU with a polyhistidine tag at the C-terminus was performed using *E. coli* strain C41(DE3) carrying pVP67K plasmid encoding IscU-His_8_. Expression of IscU was induced by addition of 1 mM IPTG at *A*600 = 0.6. After 3 h, cells were harvested and lysed in a French Press in buffer L (25 mM Tris-HCl, pH 8.0, 10% glycerol, 1 mM PMSF, 150 M KCl) containing 20 mM imidazole, pH 8.0. After a clarifying spin, the supernatant was loaded on a 2-ml Ni-NTA (Novagen (EMD Millipore Corp.), Billerica, MA, USA) column equilibrated in buffer L. After wash steps with buffer L and buffer L with 1 M KCl, proteins were eluted with a 20–500 mM imidazole gradient in buffer L. Fractions containing IscU, which were 95% pure, were collected and concentrated by ultrafiltration prior to buffer exchange to buffer F (20 mM Tris-HCl pH 8.0, 20 mM KCl, 10% glycerol) using a PD10 column (GE Healthcare, UK). Final preparations of IscU were aliquoted and stored at −70 °C.

Expression of IscU-GST was induced in the *E. coli* strain C41(DE3) carrying the pET3aIscU-GST plasmid by addition of 1 mM IPTG at A_600_ = 0.6. After ~16 h, cells were harvested and lysed in a French press in buffer I (25 mM Tris–HCl, pH 8.0, 200 mM NaCl, 1 mM PMSF, 1mM DTT, 10% glycerol, and 0.05% Triton X-100). After a clarifying spin, the supernatant was loaded on a 1 mL glutathione agarose column (Sigma-Aldrich, USA) equilibrated with 10 volumes of buffer I. Next, the column was washed with 100 mL of buffer I and with 10 volumes of buffer I with 0.5 M NaCl and with 10 volumes of buffer I with 10 mM MgCl_2_ and 1 mM ATP. After a final wash with 10 volumes of buffer I, proteins were eluted with buffer E (25 mM Tris–HCl, pH 8.0, 200 mM NaCl, 1 mM DTT, 10% glycerol, 0.05% Triton X-100, and 50 mM reduced glutathione). Fractions containing IscU-GST were pooled, concentrated through ultrafiltration, and passed over a PD10 column (GE Healthcare, UK) in order to change buffer to buffer G (20 mM Tris–HCl, pH 8.0, 10% glycerol, 5 mM β-mercaptoethanol, 0.05% Triton X-100, and 200 mM KCl), and stored at −70 °C

Yeast Hsc20_His_ and Mge1_His_ were purified according to [32]. Ssq1 was purified as described in [47]. Recombinant Isu1 with a polyhistidine tag at the C-terminus, used for the ATPase assays, and Isu1-GST fusion protein, used for the pull-down assay were purified as described previously in [23] and [48], respectively. *Chaetomium termophilum* CtIsu1 with a polyhistidine tag at the C-terminus was purified according to [19]. In all cases, protein concentrations, determined using the Bradford (Bio-Rad Laboratories GmbH, München, Germany) assay system with bovine serum albumin as a standard, are expressed as the concentration of monomers.

### 4.3. ATPase Activity of Hsp70

#### 4.3.1. Radioactive ATPase Assay

ATPase activity was measured as described by [23] with 0.5 μM HscA, 10 μM IscU, and HscB at the indicated concentrations or with 0.5 μM Ssq1, 10 μM Isu1, 0.5 μM Mge1, and Hsc20 at the indicated concentrations in buffer A (40 mM 4-(2-hydroxyethyl)-1-piperazineethanesulfonic acid (HEPES–KOH), pH 7.5, 100 mM KCl, 1 mM dithiothreitol, 10 mM MgCl_2_, and 10% (*v*/*v*) glycerol). Reactions (15 μL) were initiated by the addition of ATP (2 μCi, 2200 Ci/mmol, HARTMANN ANALYTIC GmbH, Braunschweig, Germany) to a final concentration of 120 μM. Incubation was carried out at 25 °C, and the reaction was terminated after 15 min by the addition of 100 μL of 1 M perchloric acid and 1 mM sodium phosphate. After addition of 400 μL of 20 mM ammonium molybdate and 400 μL of isopropyl acetate, samples were mixed and the phases were separated by a short centrifugation. An aliquot of the organic phase (150 μL), containing the radioactive orthophosphate-molybdate complex, was removed and radioactivity was determined by liquid scintillation counting. Control reactions lacking protein were included in all experiments. Values were plotted in GraphPad Prism 7 (version 7.02, GraphPad Software, San Diego, CA, USA) using the Michaelis–Menten hyperbolic equation to fit the data. 

#### 4.3.2. Enzyme-Coupled Spectrophotometric Assay

Steady-state ATPase assay was performed as described previously [49].To determine the Km_ATP_ values for HscA and Ssq1, reaction mixtures containing 1 μM HscA or 2.5 μM Ssq1 and 2.5 μM Mge1, buffer CA (50 mM HEPES-KOH, pH 7.5, 150 mM KCl, 20 mM magnesium acetate, 1 mM dithiothreitol), 0.265 mM NADH, 100 U/mL lactic dehydrogenase (LDH), 70 U/mL pyruvate kinase (PK), 2.8 mM phosphoenolpyruvate (PEP) and indicated concentrations of ATP. Reactions were initiated by mixing of ATP with proteins and incubated at 23 °C. NADH absorbance was measured at 340 nm for 500 s in a JASCO V-660 UV-Vis spectrophotometer. 

#### 4.3.3. EnzChek Phosphate Assay

The ATPase activity of HscA or Ssq1 (0.5 μM ) in the presence and absence of excess IscU/CtIsu1(50 μM) and HscB/Hsc20 (50 μM) was assessed at 23 °C by measuring the phosphate released via a coupled enzyme assay [50] with the EnzChek Phosphate Assay Kit (Molecular Probes) using JASCO V-660 UV-Vis spectrophotometer. For Ssq1, Mge1 was also added (0.5 μM). The ATPase rates were determined in HKM buffer (50 mM HEPES-KOH, pH 7.5, 150 mM KCl, 5 mM MgCl_2_, 1 mM dithiothreitol) in the presence of 1 mM ATP.

### 4.4. GST- Pull-Down Assay

To determine HscA and Ssq1 affinities for protein substrates, pull-down experiments were performed as described in [32] in the presence of ATP or ADP (4 mM), using IscU-GST and Isu1-GST. It was shown previously that Isu1-GST can replace native Isu1 in *S. cerevisiae in vivo* [51] In a total of 150 μL reaction volume, 30 μM IscU-GST/Isu1-GST was incubated with increasing concentration of HscA/Ssq1 in PD buffer (40 mM HEPES–KOH, pH 7.5, 100 mM KCl, 1 mM dithiothreitol, 10 mM MgCl_2_, 5% [*v*/*v*] glycerol). Bacterial proteins were incubated for 30 min at 30 °C and yeast proteins 15 min at 25 °C to allow complex formation. Reduced glutathione immobilized agarose beads (Sigma-Aldrich, USA) were pre-equilibrated with 0.1% bovine serum albumin, 0.1% TritonX-100, and 10% (*v*/*v*) glycerol in PD buffer. 40 μL of bead suspension (~20 μL bed volume) was added to each reaction and incubated at 4 °C for 1 h with rotation. The beads were washed one time with 500 μL of PD buffer and then three times with 200 μL of PD buffer. Proteins bound to the beads were incubated with 20 μL of 4-fold concentrated Laemmli sample buffer for 10 min at 100 °C and aliquots were loaded on SDS-PAGE and visualized by Coomassie blue staining. To measure HscA and Ssq1 affinities for HscB and Hsc20 cochaperones, IscU-GST (30 μM) was incubated with HscA (30 μM) in the presence of ATP (4 mM) and increasing concentrations of HscB and Isu1-GST (2.5 μM) was incubated with Ssq1 (4 μM) in the presence of ATP (4 mM) and increasing concentrations of Hsc20. After a 30 min incubation for bacterial proteins at 30 °C and 15 min at 25 °C for yeast proteins, glutathione resin was added to pull-down GST and associated proteins. After the wash steps with PD buffer, proteins bound to the beads were incubated with 20 μL of 4-fold concentrated Laemmli sample buffer for 10 min at 100 °C and aliquots were loaded on SDS-PAGE and visualized by Coomassie Blue staining. Every experiment was repeated three times and after quantitation by densitometry, average values were plotted with their respective standard deviations. The Michaelis–Menten hyperbolic equation was fit to the data using GraphPad Prism software (version 7.02, GraphPad Software, San Diego, CA, USA).

### 4.5. Circular Dichroism

Measurements of circular dichroism spectra were performed on a Jasco J-1500 CD Spectrometer from 197 to 260 nm with a 1 nm step size at 20 °C. The protein concentration was 5 μM for HscB, HscA and Hsc20, and 2.5 μM for Ssq1. Measurements were performed in buffer CD (20 mM potassium phosphate pH 8.0, 80 mM KCl) in a quartz cuvette with 1-mm path length. Spectra were measured in millidegrees (mdeg), corrected for buffer effects, and converted to mean residue ellipticity (Θ).

Melting temperatures (T_M_) of proteins were measured as follows. A wavelength corresponding to the minimum, 220 nm for HscB, 218.4 nm for HscA, 222.7nm for Hsc20 and 220.4 nm for Ssq1, was used to monitor the thermal melting, expressed as a change in mdeg as the temperature increased from 20 to 85 °C at a rate of 0.5 °C/min. Data were analysed using Spectra Manager ver. 2, JASCO software in order to calculate the T_M_ values for each protein.

## Supplementary Materials

The following are available online at https://www.mdpi.com/1422-0067/21/9/3326/s1.
