# Supplementary figures and images for: Biochemical Convergence of Mitochondrial Hsp70 System Specialized in Iron–Sulfur Cluster Biogenesis
Source: Int J Mol Sci. 2020 May 8;21(9):3326. doi: 10.3390/ijms21093326 (PMC7247549; doi:10.3390/ijms21093326)

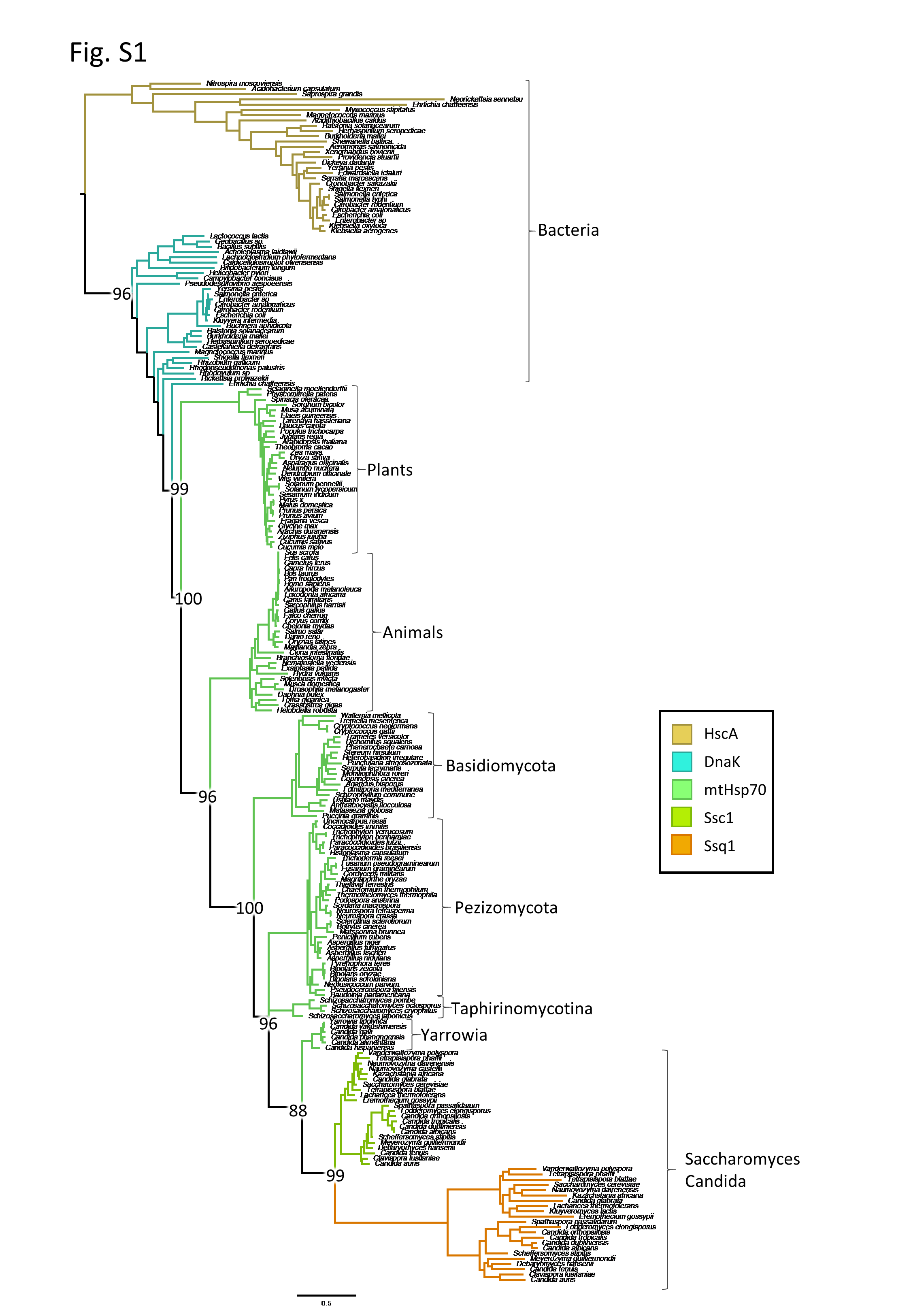

Supplement: Supplementary file 1 [file ijms-21-03326-s001.zip › Supplementary_Fig_Revision final/Slajd1.TIF]

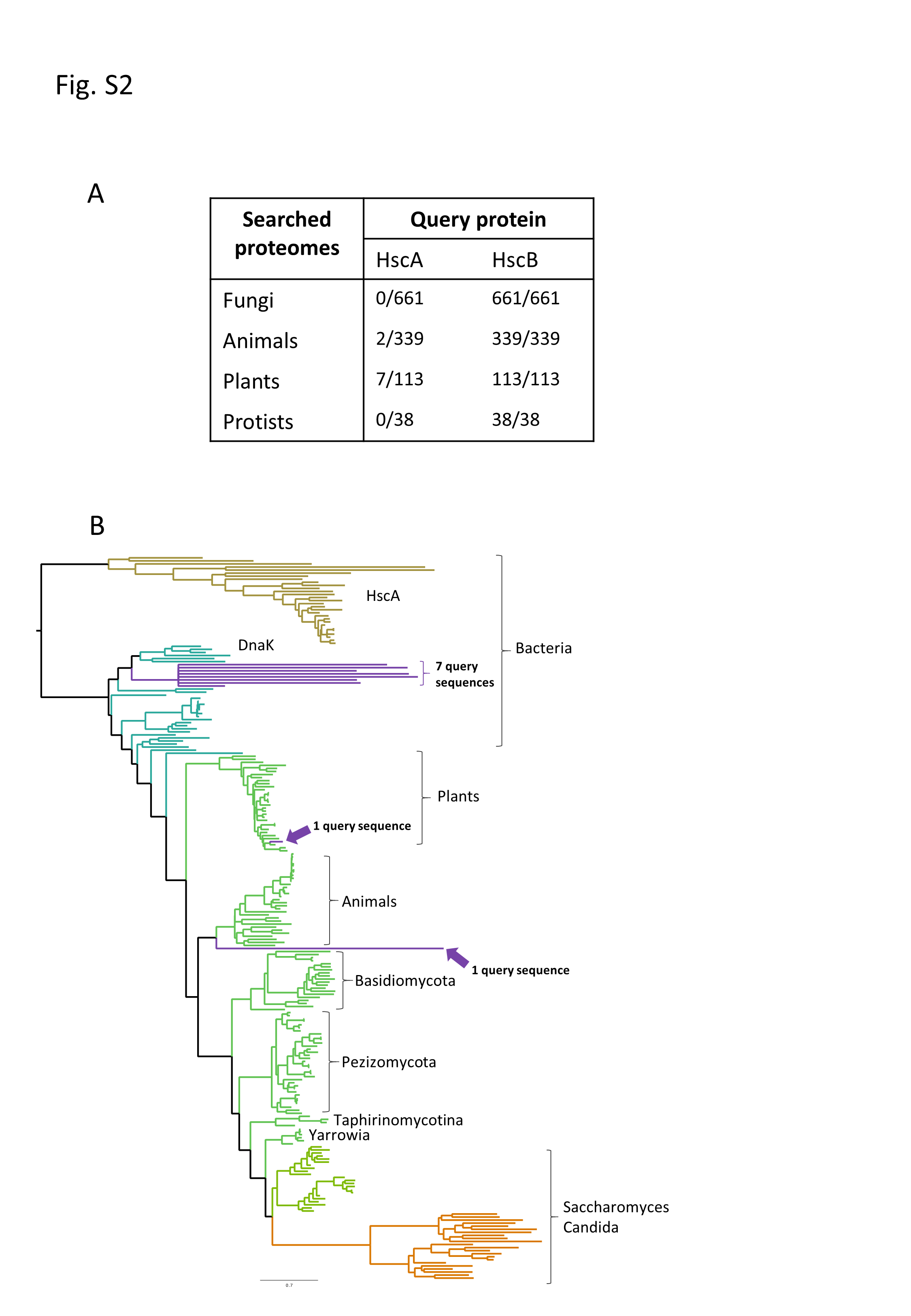

Supplement: Supplementary file 1 [file ijms-21-03326-s001.zip › Supplementary_Fig_Revision final/Slajd2.TIF]

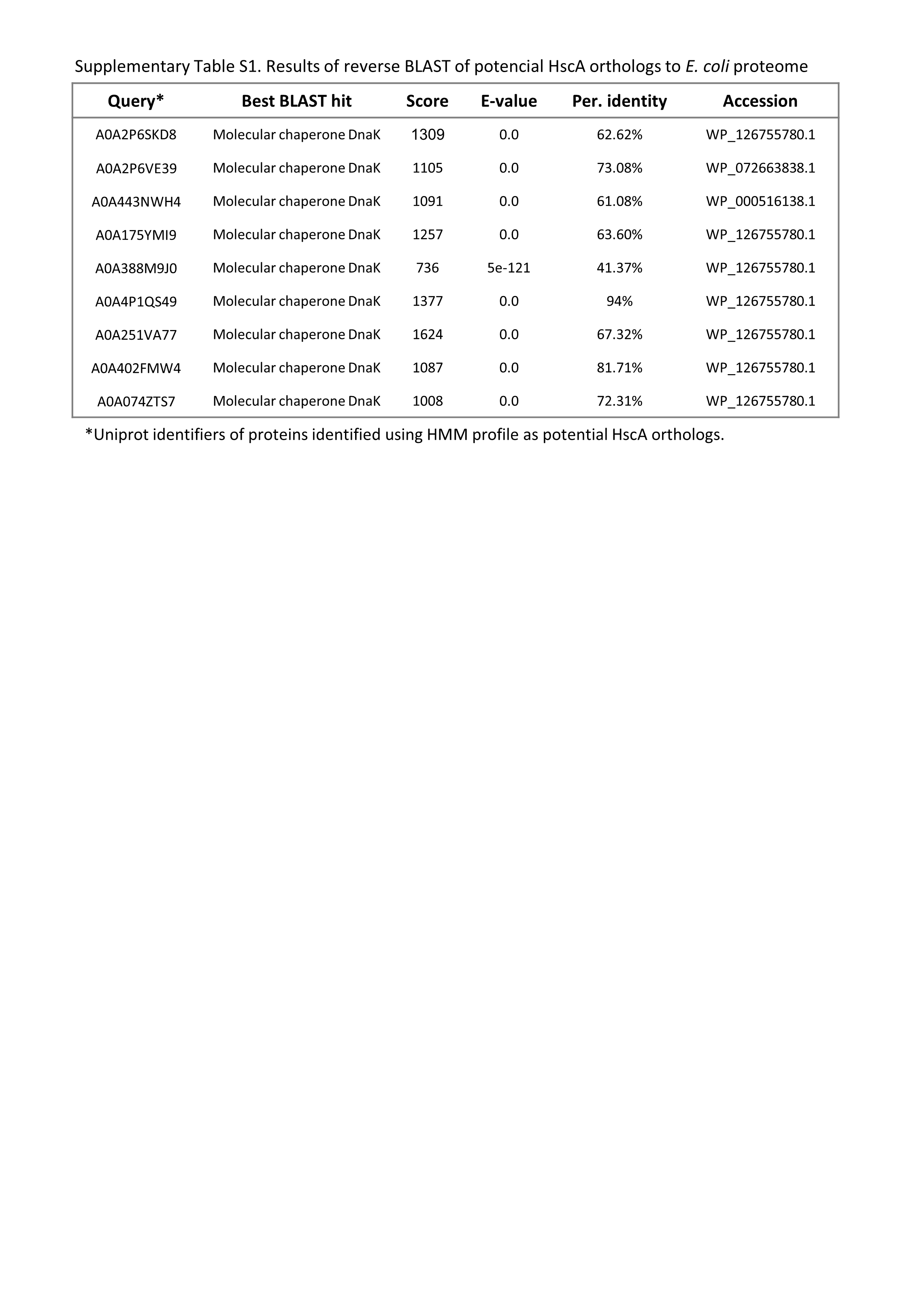

Supplement: Supplementary file 1 [file ijms-21-03326-s001.zip › Supplementary_Fig_Revision final/Slajd3.TIF]

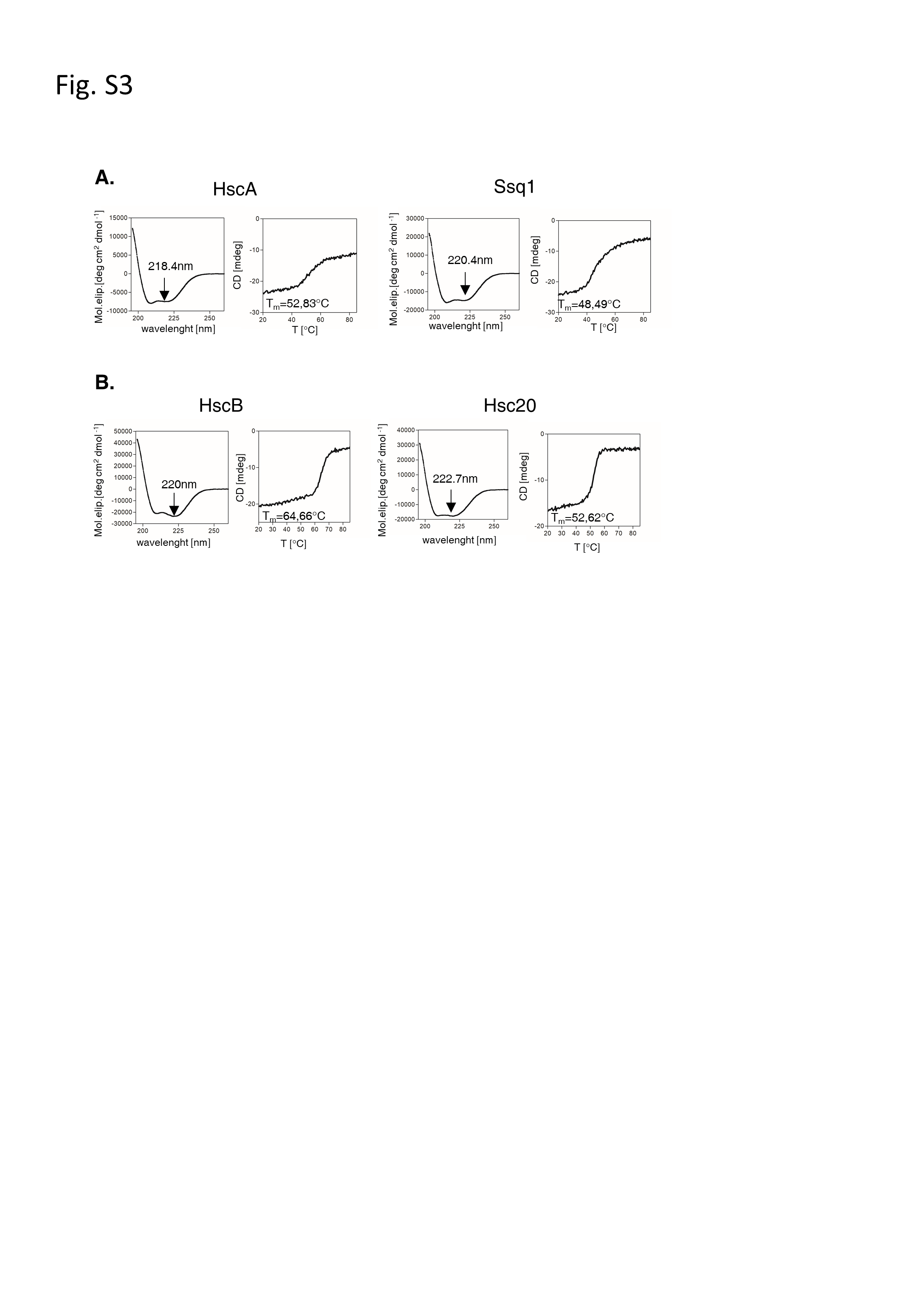

Supplement: Supplementary file 1 [file ijms-21-03326-s001.zip › Supplementary_Fig_Revision final/Slajd4.TIF]

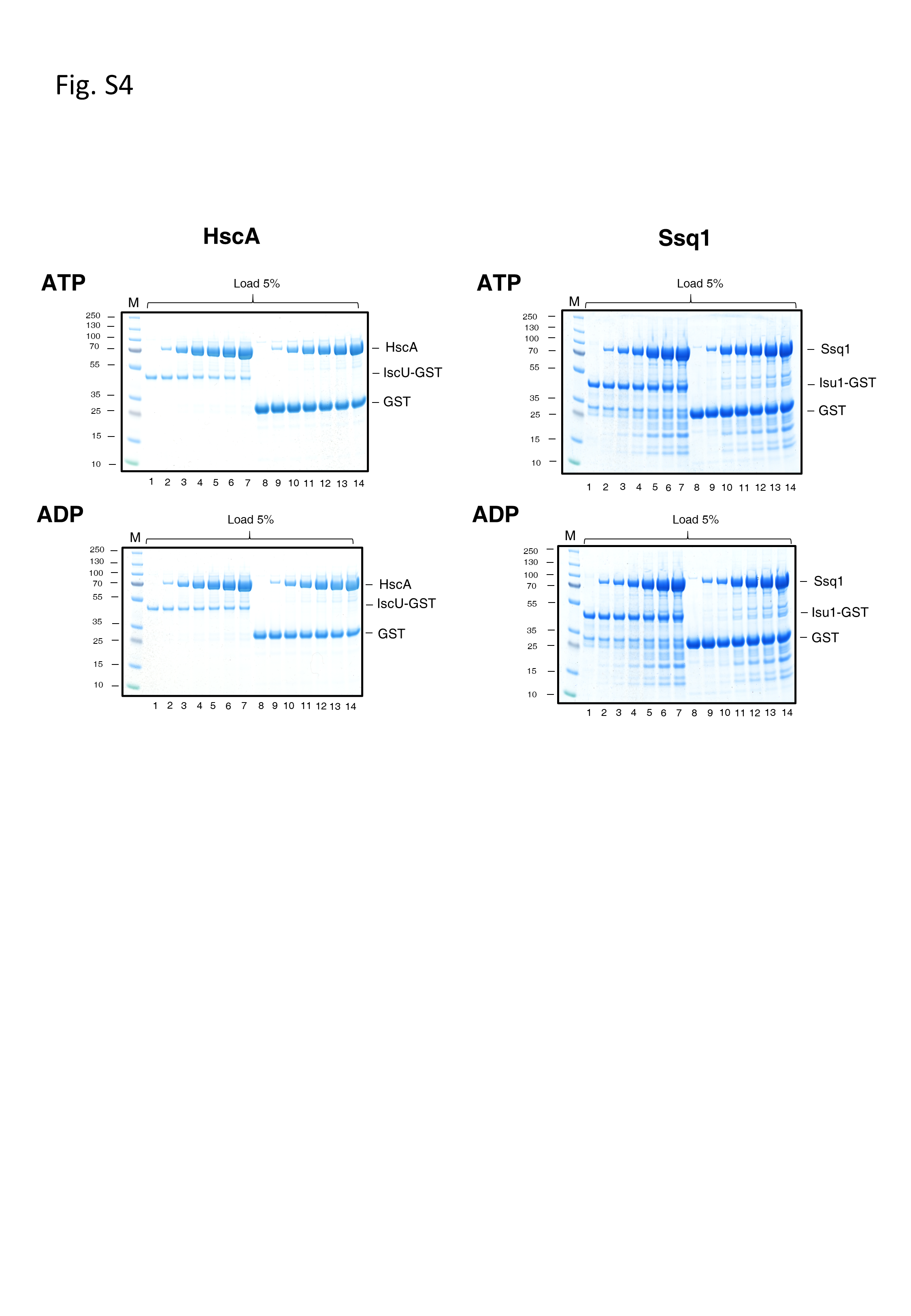

Supplement: Supplementary file 1 [file ijms-21-03326-s001.zip › Supplementary_Fig_Revision final/Slajd5.TIF]
